# Multi-morbidity of non communicable diseases and equity in WHO Eastern Mediterranean countries

**DOI:** 10.1186/1475-9276-12-60

**Published:** 2013-08-20

**Authors:** Abdesslam Boutayeb, Saber Boutayeb, Wiam Boutayeb

**Affiliations:** 1 Department of Mathematics, LaMSD and URAC04, Faculty of Sciences, University Mohamed Ier, Oujda, Morocco; 2 Department of medical oncology, National Institute of Oncology, Rabat, Morocco

## Abstract

**Introduction:**

Non communicable diseases are the biggest cause of death worldwide. Beside mortality, these diseases also cause high rates of morbidity and disability. Their high prevalence is generally associated to multi-morbidity. Because they need costly prolonged treatment and care, non communicable diseases have social and economical consequences that affect individuals, households and the whole society. They raise the equity problem between and within countries.

**Methods and limitations:**

This annotated bibliography is a systematic review on multimorbidy of non communicable diseases and health equity in WHO Eastern Mediterranean countries. Medline/PubMed, EMBASE and other sources were used to get peer reviewed papers dealing with the review theme. The words/strings used for search and inclusion criteria were: multimorbidity, comorbidity, equity, non communicable diseases, chronic diseases, WHO Eastern Mediterranean and Arab countries.

**Bibliography with annotations:**

According to the inclusion criteria, 26 papers were included in the present review. Generally, lack or paucity of publications was encountered in themes like headache, cancer and respiratory diseases. Of the 26 contributions selected, twelve dealt with comorbidity of depression and mental disorders with other chronic diseases. Another set of 11 publications was devoted to multimorbidity of diabetes, cardiovascular diseases (CVDs), hypertension, metabolic syndrome and obesity. Considering association of multimorbidity and social determinants, this review shows that female gender, low income, low level of education, old age and unemployed/retired are the most exposed to multimorbidity. It should also be stressed that, geographically, no contribution was issued from North African countries.

Non communicable diseases are one of the biggest challenges facing health decision makers in WHO Eastern Mediterranean countries where the multidimensional transition is boosting increases in multimorbidity of depression and mental diseases, cardiovascular diseases, diabetes, cancer and respiratory diseases among the whole population but with the highest burden among the least disadvantaged individuals or subpopulations. Health ministries in WHO Eastern Mediterranean countries should pay a particular attention to the association between equity and multimorbidity and opt for cost effective strategies based on early diagnosis and sensitisation for healthy diet, physical activity, no smoking and no alcohol.

## Introduction

One of the biggest challenges currently facing humanity is chronic diseases which are sweeping the entire globe, with an increasing trend in developing countries [1]. The WHO Global status report on non communicable diseases (NCDs) 2010 showed that NCDs were globally the biggest cause of death. Of the 57 million deaths that occurred worldwide in 2008, about 36 million (63%) were due to non communicable diseases, principally cardiovascular diseases (48%), cancer (21%), chronic respiratory diseases (12%) and diabetes (4%). Unfortunately, more than 9 million of these deaths occurred before the age of 60 and could have largely been prevented [2].

Low- and middle-income countries were the home of 80% of theses NCDs. Moreover, more than 40% of NCD-related deaths in low-income countries occurred under the age of 60, nearly three times the proportion in high-income countries (13%). If the trend is not reversed or at least stopped, NCD deaths are expected to reach 44 million deaths by 2020. During the next decade, the greatest increases will occur in the WHO regions of Africa, South-East Asia and the Eastern Mediterranean (increases over 20%, compared to a global increase of 15%). Most non communicable diseases have globalization, urbanization and ageing as underlying determinants; and unhealthy diet, physical inactivity and tobacco/alcohol as common risk factors. They also share intermediate risks like high blood sugar, raised blood pressure, overweight/obesity and abnormal blood lipids. “The two major inextricably related issues, aging and chronic disease, create challenges for public health and clinical care in settings already faced with scarce recourses” [3].

Because they need costly prolonged treatment and care, non communicable diseases have social and economical consequences that affect individuals, households and the whole society. They raise the equity problem between and within countries [4].

Availability and affordability of adequate treatment is a crucial problem in low-income countries but it may also be a concern for poor populations in middle- and high-income countries. For these reasons, non communicable diseases should be considered as a sustainable development issue, striking both rich and poor people, but inflicting more ill health and other consequences on the poor in all countries [5].

The World Economic Forum 2010 enumerated 5 sets of risks that share a potential for wider systematic impact and are strongly linked to a number of significant, long-term trends: Economic, Geopolitical, Environmental, Societal and Technological risks. Out of the 36 global risks considered, chronic diseases were classified in the first group with the highest economic severity (more than 1 trillion US$) [6].

In the WHO Eastern Mediterranean Region, non communicable diseases account for 53% of annual mortality with 2.3 million deaths per year. Nearly 1.2 million deaths were caused by cardiovascular diseases (CVDs), representing 55% of deaths caused by NCDs and 28.5% of all deaths in the region. The regional distribution range is, however, very wide (from 13% in Somalia to 49% in Oman). Cancer caused 14% of NCD-related deaths and 7.5% of all deaths in the region but, again, figures vary from a minimum of 3% in Afghanistan and Somalia to a maximum of 20% in Qatar. In opposition, the percentage of deaths caused by respiratory diseases are homogenous in the whole region, ranging from 2% to 5% of total deaths (Table 1) [7].

**Table 1:** Percentage of deaths caused by non communicable diseases in the WHO Eastern Mediterranean Region

**Countries**	**Non communicable diseases (%)**					
	**CVD**	**Cancer**	**Respiratory**	**Diabetes**	**Other NCDs**	**Total NCDS**
Afghanistan	15	3	2	1	9	**29**
Bahrain	32	12	5	12	18	**79**
Djibouti	21	5	2	1	12	**42**
Egypt	39	11	3	3	26	**82**
Iran	45	12	4	2	10	**72**
Iraq	25	7	2	1	9	**44**
Jordan	40	11	3	7	13	**74**
Kuwait	46	13	2	3	12	**76**
Lebanon	45	19	5	2	13	**84**
Libya	43	13	4	2	15	**78**
Morocco	40	12	5	2	16	**75**
Oman	49	11	3	7	13	**83**
Pakistan	25	7	5	1	8	**46**
Qatar	23	20	4	7	15	**69**
Saudi Arabia	42	9	3	6	12	**71**
Somalia	13	3	2	1	8	**27**
Sudan	23	4	3	2	11	**44**
Syria	44	8	4	3	18	**77**
Tunisia	38	16	4	1	12	**72**
UAE	38	12	2	3	11	**67**
Yemen	24	5	3	1	12	**45**
**% of total deaths**	**28.5**	**7.5**	**3.8**	**1.7**	**11.6**	**53.1**
**Total deaths in EMR= 4,198,000**						
	**1195**	**315**	**159**	**71**	**489**	**2229**
**% of NCDs**	**55%**	**14%**	**9%**	**5%**	**17%**	**100%**

According to the data released by the International Diabetes Federation (IDF) in 2011, the Middle East and North Africa region has the highest comparative prevalence of diabetes (11%). Six of the top 10 countries with the highest prevalence of diabetes (in adults aged 20 to 79 years) are in this region: Kuwait (21.1%), Lebanon (20.2%), Qatar (20.2%), Saudi Arabia (20.0), Bahrain (19.9%) and UAE (19.2%) [8,9]. Diabetes-related deaths occurring in the WHO EMR countries range from 1% in seven countries to 12% in Bahrain [7].

During the last decade, the focus on health equity has grown in importance. In 2005, the World Health Organization established the WHO Commission on Social Determinants of Health which delivered its interesting report in 2008 under the title “Closing the gap in a generation: health equity through action on the social determinants of health”. The report stated that “The social determinants of health are the conditions in which people are born, grow, live, work and age, including the health system. These circumstances are shaped by the distribution of money, power and resources at global, national and local levels. The social determinants of health are mostly responsible for health inequities - the unfair and avoidable differences in health status seen within and between countries” [10].

Another illustration of equity is provided by the 2010 human development report, introducing a new human development index (HDI) which captures the losses in human development due to inequality in health, education and income, namely: the Inequality-adjusted HDI (IHDI) [11]. In 2011, losses in the three dimensions varied across countries, ranging from 1 percent in education (Czech Republic) to 68 percent in income (Namibia). The WHO Eastern Mediterranean countries Djibouti, Egypt, Jordan, Lebanon, Morocco, Pakistan and Syria lost respectively, 35.9%, 24.1%, 19%, 22.8%, 29.7%, 31.4% and 20.4% of their human development due to inequality.

Since the last report released by the WHO Commission on Social Determinants of Health in 2008 and the Rio Political Declaration on Social Determinants of health adopted by heads of government, ministers and government representatives in October 2011, health equity should become part of a government’s social and health agenda. It is to be a shared responsibility requiring the engagement of all sectors of governments, and all national and international agencies in “an all-for-equity” global action [12]. Health equity has, however, been less associated with multi-morbidity, especially in developing countries. This paper is devoted to multi-morbidity and equity in the WHO Eastern Mediterranean Region.

## Methods and limitations

### Database sources

Firstly, Medline/PubMed and EMBASE and other sources were used to get peer reviewed papers dealing with the review theme. Secondly, websites of peer-reviewed journals and well known organisations like World Health Organisation, International Diabetes Federation, World Heart Federation, Eastern Ministries of health and other sources were browsed to obtain complementary information. Thirdly, references given in the papers selected through steps one and two (especially those provided by systematic reviews) were also scrutinised.

### Keywords and time interval used for data extraction

The words/strings used were: multimorbidity, multi-morbidity, comorbidity, co-morbidity, equity, non communicable diseases, chronic diseases, Eastern Mediterranean, Arab and name of countries belonging to the WHO Eastern Mediterranean Region (WHO-EMR). The search was limited to publications between 1992 and 2012(30^th^ September).

### Inclusive/exclusive criteria

For a paper to be included in the review, five criteria were jointly required 1) Chronic/non communicable diseases, 2) Multimorbidity/comorbidity, 3) WHO Eastern Mediterranean region, 4) health equity, measured by socioeconomic status, education level, gender, ethnicity, etc.…and 5) Availability of abstract. When more than a paper was selected with the same authors and the same topic, only one paper was included in the review.

### Presentation

For each paper cited, using a slightly different and concise form of available abstract, a summary of the method is given with the main results on mutimorbidity and equity. The papers reviewed were grouped into sub topics (Table 2): **1)** Cancer, **2)** Respiratory diseases, **3)** Depression, mental disorders and NCDS and **4)** Diabetes, CVDs, Metabolic Syndrome, hypertension and obesity.

**Table 2:** Publications by author, title, multimorbidity and social determinants

**Author (date of publication)**	**Title**	**Country/region**	**Size of sample**	**Mutimorbidity**	**Equity, social determinants**
[13] Aziz & Sana Gan To Kagaku Ryoho 2002, 29(1):4-8	Cancer treatment in Pakistan: challenges & obstacles.	Pakistan	3274	Cancer, hepatitis B & C	Age, sex, socioeconomic status, occupation
[14] Riachy et al. *Revue des maladies Respiratoires* 2008, 25(3):275-281	Impact of low socioeconomic status on the demography and co-morbidities of asthma.	Lebanon	44814	Asthma, obesity, depression ocular and cutaneous allergy	Age, sex, income, education, housing
[15] Aslani et al. Tanaffos 2007, 6(2):38-45	The Socioeconomic Status and Quality of Life in Patients with Chronic Obstructive Pulmonary Disease.	Pakistan	131	Poor pulmonary function, respira-tory symptoms, incapacity to perform daily activities, mental and cognitive disorders.	Age, sex, education, marital status
[16] Kilzieh et al. (Int J Psychatry Med 2008, 38(2):169-184	Comorbidity of depression with chronic diseases: a population-based study in Aleppo, Syria	Aleppo, Syria	2038	Depression chronic respiratory disease, heart disease, hypertension, stroke, diabetes, Rheumatism, Peptic ulcer, kidney disease, hepatic disease and obesity	age, residence, ethnicity, Sex, religion, education occupation, marital status socio-economic status
[17] Moussavi et al. (The Lancet 2007, 370:851–858)	Depression, chronic diseases, and decrements in health: results from the WHS	Morocco	4472	Depression, angina, arthritis, asthma and diabetes	Sex, age, residence, education, occupation, marital status, income
		Pakistan	6104		
		Tunisia	5068		
		UAE	1180		
[18] Hosseinpoor et al. BMC Public Health 2012, 12:474	Socioeconomic inequality in the prevalence of NCDs in LMICs: Results from the World Health Survey	Morocco	4472	Depression, angina, arthritis, asthma and diabetes	Sex, age, residence, education, occupation, marital status, income
		Tunisia	5068		
		Pakistan	6104		
[19] Khuwaja et al. Diabetology & Metabolic Syndrome 2010, 2:72	Anxiety and depression among outpatients with type 2 diabetes: A multi-centre study of prevalence and associated factors.	Pakistan	889	Depression, anxiety, hypertension, ischemic heart disease	Age, sex
[20] Yekta et al. Eastern Mediterranean Health Jornal 2010, 16:286–291.	Behavioural and clinical factors associated with depression among individuals with diabetes.	Urmia, Iran	295	Depression, diabetes	Age, sex, education
[21] Al-Amer et al. Journal of Diabetes and its complications 2011, 25:247-252	Depression among adults with diabetes in Jordan risk factors and relationship to blood sugar control	Jordan	649	Depression, diabetes, hypertension	Age, sex, education
[22] Waheed et al. Journal of the Pakistani Medical Association 2006, 56:243	The Burden of Anxiety and Depression among patients with Chronic Rheumatologic Disorders at a Tertiary Care Hospital Clinic in Karachi, Pakistan.	Pakistan	111	Depression, anxiety, chronic rheumatological disorders	Age, sex, education, occupation, income
[23] Youssef Eastern Mediterranean Health Journal 2005,11(3):334-348	Comprehensive health assessment of senior citizens in Al-Karak governorate, Jordan	Al-Karak Jordan	300	Mental chronic diseases	Age, sex, education, Marital status
[24] Bener et al. Social Psychiatry Epidemiolofy 2012, 47(3):439-446	Prevalence, symptom patterns and comorbidity of anxiety and depressive disorders in primary care in Qatar	Qatar	1660	Depression, anxiety, diabetes, hypertension, asthma, back pain, cancer, migraine, cholesterol	Sex, age, education, occupation, marital status, household income, housing
[25] Ohaeri et al. *Medical Science Monitoring* 2012, 18(3):CR160-73	Characteristics of subjects with comorbidity of symptoms of generalised anxiety and major depressive disorders and the corresponding threshold and sub-threshold conditions in an Arab general population sample.	Kuwait	3155	Depression anxiety	Age, sex, education, marital status, occupation
[26] Shiri et al. The American Journal on Addictions 2006, 15(6):468-472	Association between Opium Abuse and Comorbidity in Diabetic Men.	Pakistan	312	Diabetes, mental disorders, opium	Age, socio-economic status
[27] Almawi et al. Journal of Endocrinollogical Investigation. 2008, 31(11):1020-1024	Association of comorbid depression, anxiety, and stress disorders with Type 2 diabetes in Bahrain, a country with a very high prevalence of Type 2 diabetes	Bahrain	275	Diabetes, depression, anxiety	Age, sex, occupation,
[28] Shah et al. Medical Principles and Practice 2010, 19(2):105-112	Prevalence and correlates of major chronic illnesses among older Kuwaiti nationals in two governorates	Kuwait	2487	Diabetes, hypertension, heart disease	Age, sex, education, occupation, income,
[29] Alwakeel et al. Saudi Journal of Kidney Diseases and Transplantation 2009, 20(3):402-409	Concomitant macro and microvascular complications in diabetic nephropathy.	Saudi Arabia	1952	Diabetes, macrovascular, Microvascular complications	Age, sex
[30] Shera et al. Journal of the Pakistan Medical Association 2004, 54(2):54-59	Prevalence of Chronic Complications and Associated Factors in Type 2 Diabetes	Pakistan	500	Diabetes, retinopathy, nephropathy, neuropathy, angina, hypertension	Age, sex,
[31] Maziak et al. Ann Epidemiol 2007, 17:713–720.	Cardiovascular health among adults in Syria: a model from developing countries.	Syria	2028	Heart disease, stroke, hypertension, obesity	age, sex, education, occupation, income and household density
[32] Khan et al. *BMC Cardiovascular Disorders* 2006, 6:18	Knowledge of modifiable risk factors of heart disease among patients with acute myocardial infarction in Karachi, Pakistan: across sectional study	Karachi, Pakistan	720	Heart disease, diabetes, hypertension	.
[33] Zindah et al. *Prev Chronic Dis* 2008, 5(1):A17	Obesity and diabetes in Jordan: findings from the behavioral risk factor surveillance system, 2004.	Jordan	3334	Obesity, diabetes, heart disease, asthma, hypertension,	Age, sex, education, socio demographic
[34] Khader et al. Metabolic Syndrome and Related Disorders 2008, 6(2):113-120	Obesity in Jordan: Prevalence, Associated factors, comorbidities, and change in prevalence over the ten years	North Jordan	1121	Obesity, diabetes, hypertension, low HDL, hypertriceridemia	gender, age, marital status, education
[35] Abdul-Rhaim Int J Obes 2001, 25:1736-1740	Obesity and selected comorbidities in an urban Palestinian population.	Ramallah Palestine	485	Obesity, diebetes, Hypertension, cholesterol	Age, sex, education, smoking
[36] Delavar et al. Asia Pac J Clin Nutr 2009, 18 (2):285-292	Dietary patterns and the metabolic syndrome in middle aged women, Babol, Iran.	Babol, Iran	984	Obesity, metabolic syndrome	Age, sex, education, occupation
[37] Hollisaaz et al. Transplantation Proceedings 2007, 39(4):1048-1050	Medical Comorbidities after Renal Transplantation	Pakistan	119	Hypertension, visual disturb, Back pain, musculoskeletal	Age, sex, marital status, education, income
[38] Bernieh et al. Transplantation Proceedings *2004* 36(6):1780-1783	Pattern of acute renal failure in a tertiary hospital in the United Arab Emirates	UAE	81	ARF, diabetes, hypertension, ischemic heart disease	Age, sex, ethnicity

## Bibliography with annotations

### Cancer

#### Cancer and hepatitis (Pakistan)

Aiming to determine socioeconomic status, disease stage, co-morbid conditions in a population suffering from cancer, Aziz and Sana [13] analysed demographic variables including age, sex, socioeconomic status, smoking, occupation and other co-morbid factors. They analysed a sample of 3,274 patients who attended the Department of Oncology between 1995 and 2000. All patients had histopathologically confirmed diagnosis of cancer. Poor socio-economic status was present in 89% of cases. Illiteracy was present in 76%. Comorbid conditions like hepatitis B & C were present in 37% of patients. Advanced disease was documented in 59% patients. Advanced stage, poor socio-economic status and illiteracy were common. Associated co-morbid conditions were a major cause in delay in treatment [13].

### Respiratory diseases

#### Asthma, obesity, depression (Lebanon)

Aiming to determine the demography of asthma in a low socio-economic community in Lebanon and to describe its association with various epidemiologic factors, Riachy et al. [14] considered the computerized data of 44,814 patients of a nongovernmental organization, in Lebanon. Asthmatic patients diagnosed by a health professional on the basis of medical criteria during the period from January 2003 to June 2005 were included in the analysis. Socio-economic characteristics and their geographical distributions were considered. The focus was on the association of asthma with cutaneous and ocular allergies, depression, obesity and alcohol consumption. The authors found that 583 (1.3%) of patients on the database were asthmatic and they belonged in majority (75%) to a low socio-economic environment with a salary lower than $200 per month. Nearly a third of them (31%) were illiterate. The rate in children was higher (2.08%) than in adults (1.09%). The majority of asthma occurred among subjects from Bekaa valley and South Lebanon. Asthma was associated more strongly with being an ex-smoker (OR 4.37) than being a current smoker (OR 1.44). A significant and strong association was found with depression (OR 25.6), obesity (OR 4.09) and with regular alcohol consumption (OR 11.78) [14].

### COPD (Pakistan)

In a case–control study (131 subjects), Aslani et al. [15] considered the association between chronic obstructive pulmonary disease (COPD) with poor pulmonary function, respiratory symptoms, incapacity to perform daily activities, as well as mental and cognitive disorders. They investigated the association between income and quality of life in COPD patients. Subjects were divided into three groups based on their household monthly income and the groups were matched with regard to gender, age, educational background, marital status, comorbidity burden, and insurance coverage. The overall quality of life and physical health subscale were found to be significantly different between the groups. The authors concluded that quality of life and physical function of COPD patients were significantly correlated with their socioeconomic status [15].

### Depression and NCDS

#### Depression and chronic diseases (Aleppo, Syria)

A sample of 2038 people aged 18–65 years was considered in a cross-sectional study conducted in Aleppo, Syria in order to study the comorbidity of depression with the following chronic diseases: chronic respiratory disease, heart disease, hypertension, stroke, diabetes, rheumatism, peptic ulcer, kidney disease, hepatic disease and obesity. Socio-demographic variables considered were: age group, residence (formal, informal (built without approval from municipal authorities)), ethnicity (Arab, non-Arab), religion (Muslim, non Muslim), education level, occupation, marital status and socio-economic status [16]. The authors found that, in women, predictors of depression were heart disease (OR=3.95), hypertension (OR=2.92) and kidney disease (OR=2.96). Depression comorbidity with any chronic disease decreased in higher socio-economic status (middle vs low: OR=0.28; high vs low OR: 0.20). In men, predictors of depression were rheumatism (OR=7.10), and respiratory disease (OR=3.77). Depression comorbidity decreased in residence in formal zones (OR=0.22).

### Depression, angina, arthritis, asthma and diabetes (WHS)

Analyzing data from the WHO-World Health Surveys (WHS) concerning 16824 participants from four WHO Eastern Mediterranean countries (Morocco, Pakistan, Tunisia and United Arab Emirates (UAE)) among other countries, a study was carried out to determine decrements in health caused by the co-morbidity of depression with angina, arthritis, asthma and diabetes. The analysis showed that having depression was associated with the lowest health scores, either alone or co-morbid with other chronic diseases. The authors noted that co-morbidity of depression with diabetes causes even greater decrements in health than the addition of the two conditions separately. Depression alone had more negative effect than having more than two chronic diseases without depression. Having depression with two or more chronic diseases had the lowest health scores of all the disease groups. Mean health scores were examined according to socio-demographic variables, indicating that being older, having less education, having lower income and being unemployed were all indicative of decreased health status. Analysis showed also that women had a lower overall health score, and the decrements in health were greater for women who were unemployed, less educated, or widowed [17].

### Depression, angina, arthritis, asthma and diabetes (WHS)

Using the same data source (World Health Surveys), a recent study focused on data from low- and middle-income countries (LMICs). The study included data of 15644 respondents (7820 men and 7824 women) from three WHO Eastern Mediterranean countries (Morocco, Pakistan and Tunisia) amongst a total sample of 170,298 respondents from 41 LMICs belonging to the six WHO regions. The authors analysed data for angina, arthritis, asthma, depression, and diabetes. Overall, angina showed the highest prevalence rate and age-standardized prevalence tended to be elevated in women. Arthritis demonstrated the weakest inequality and diabetes prevalence was positively associated with increasing wealth quintile. Co-morbidity prevalence showed an inverse association with wealth quintile (2.5 times more prevalent in the poorest men of study than in the richest and around 1.5 times more prevalent in the poorest women than in the richest). Depression was twice as prevalent among women with no formal education as women with college/university education. The strongest education-related regular inequality was found among populations for angina [18].

### Anxiety, depression and diabetes (Pakistan)

Stressing that anxiety and depression contribute to poor disease outcomes among individuals with diabetes, Khuwaja et al. [19] carried out a study to assess the prevalence of anxiety and depression and to identify their associated factors including metabolic components among people with type 2 diabetes. Some 889 adults with type-2 diabetes were included in a cross-sectional, multi-center study in four out-patient clinics in Karachi, Pakistan. Overall, 57.9% and 43.5% participants had anxiety and depression respectively. Physical inactivity, hypertension and ischemic heart disease were found to be independently associated with anxiety. Whereas, being female, of older age, having hypertension and ischemic heart disease were found to be significantly associated with depression. Metabolic components found to be independently associated with both anxiety and depression, were systolic blood pressure, fasting blood glucose and fasting blood triglycerides. Body mass index was independently associated with depression but not with anxiety.

### Diabetes and depression (Iran)

Comorbidity of diabetes and depression was considered in a cross-sectional study devoted to patients attending a diabetes clinic in Urmia, Iran [20]. Of 295 patients, 128 (43.4%) had depression scores (≥ 15) on the Beck Depression Inventory. The mean score for all patients was 15.4. Those with depression were significantly older and less educated than those without depression, had a longer duration of diabetes and were more likely to suffer from complications. Logistic regression analysis showed that older age was the only variable significantly associated with depression [20].

### Depression, diabetes and hypertension (Jordan)

Al-Amer et al. [21] conducted a study on the comorbidity of depression and diabetes in Jordan. Analysing data of a systemic random sample of 649 type 1 and type 2 diabetic patients aged 18–75 years, the authors found that 128 (19.7%) had depression and females were more likely to develop depression than males (OR=1.91). Low-educated people were more likely to develop depression than educated people (OR=3.09) and being on insulin treatment also had a significant association with depression (OR=3.31). The authors concluded that the prevalence of depression among Jordanian subjects with type 1 and type2 diabetes was high compared with some developed countries. This was associated with gender, educational level, insulin treatment, low self-management behaviours and increased barriers to adherence [21].

### Anxiety, depression and rheumatological disorders (Pakistan)

In order to study the burden of anxiety and depression as a comorbid among patients of chronic rheumatological disorders, Waheed et al. [22] conducted a cross-sectional study at the rheumatology clinic of the Aga Khan University Hospital (AKUH) Karachi, Pakistan. The authors found that almost two third of patients with chronic rheumatological disorders also suffered from a concomitant mood disorder. The population consisted mainly of middle aged (mean age 41) females (80.2%). Marital Status, gender, economic activity and monthly family income had no effect on the frequency of anxiety and depression whereas educational qualification, permanent joint deformity, active inflammation and time elapsed since diagnosis had significant association with anxiety and depression [22].

### Mental chronic diseases (Al Karak, Jordan)

The health status, mental well-being and functional capacity of senior citizens was assessed in a community-based survey of people aged 60 years and above in 2004. Of the 300 subjects enrolled (53.3% women), 74.4% were affected by chronic diseases, 24.3% were classified as depressed and 44.0% had a negative health perception. Dependence in instrumental activities of daily living (92.0%) was more frequent than dependence in basic activities (28.0%). Women were more likely to be depressed, and suffer memory impairment and limitation of functional capacity. Increase in depressive symptoms was independently predicted by increased age, living alone, poor functional capacity, memory impairment and negative perception of health. Low summary performance in instrumental and basic activities was independently predicted by increased age, lack of education, high number of reported symptoms, depression and memory impairment. Depression, poor functional capacity and memory impairment reinforced each other resulting in a state of dependency [23].

### Anxiety, depression and NCDs (Qatar)

In 2009 a prospective cross-sectional study was conducted by Bener et al. [24] in Qatar. The aim of the study was to assess the prevalence of anxiety and depressive disorders in a Qatari population who attended the primary health care settings, and examine their symptom patterns and comorbidity. A total of 2,080 Qatari subjects aged 18–65 years were approached and 1,660 patients (46.2% males and 53.8% females) participated in the study. Socio-demographic characteristics, comorbidity factors, and medical history of patients were collected. The findings of this study revealed that depression (26.6% among males and 30.1% among females) was more prevalent in the Qatari population than anxiety disorders (18.7% among males and 24.6% among females) and women were likelier than men to suffer from depression and anxiety. Significant differences were seen between men and women with depression in terms of age group, marital status, occupation, and household income. The frequent comorbidity conditions with anxiety and depression, respectively, were: diabetes mellitus (23.4 vs. 19.2%), hypertension (25.7 vs. 25.0%), headache and migraine (21.6 vs. 25.4%), and low back pain (22.2 vs. 28.6%). The high-risk groups of depression and anxiety disorders were female gender, being married, middle aged, and highly educated [24].

### Anxiety-depression (Kuwait)

In 2006/7, comorbid generalized anxiety disorder (GAD)/major depressive disorder (MDD) was considered in a study by Ohaeri and Awadalla [25]. Amongst the 3155 Kuwaitis participants aged 16–87 years, 273 had GAD and 210 had MDD. The prevalence of comorbidity among cases with GAD was 30.8% and 40% among MDD. Comorbid threshold GAD/MDD cases were significantly older, and more likely to be women, divorced and unemployed, compared with GAD and MDD [25].

### Mental disorders, diabetes, opium (Pakistan)

A study conducted in 2005 by Shiri et al. [26] aimed to determine the prevalence of opium abuse in diabetic men and to investigate its association with comorbidity. In a sample of 312 adult men with diabetes, the self-reported prevalence of opium abuse was 11.2%. The authors reported that opium use was associated with low socioeconomic status, smoking, tea consumption, and a higher prevalence of erectile dysfunction (ED) and severe depression. The prevalence of severe depression was 22.8% among 35 men who used opium and 13.4% among 277 who did not use it. The prevalence of moderate or severe ED was 85.7% among opium users and 66.1% among non-users [26].

### Depression, anxiety, diabetes (Bahrain)

In a cross-sectional study examining the association of depression, anxiety, and stress with Type 2 diabetes (T2DM) in Bahrain, Almawi et al. [27] analysed data of 143 T2DM patients and 132 healthy controls. Higher proportion of T2DM patients were found in the mild-moderate and severe-extremely severe depression, anxiety, and stress groups. Chronic disease and disease duration were significantly associated with the 3 disturbances, while employment status was associated with anxiety and depression. Using logistic regression analysis, the authors showed that anxiety, depression, and stress were associated with T2DM after adjusting for all variables, while age was the only significant variable associated with stress [27].

### Heart disease, diabetes, hypertension, obesity, metabolic syndrome

#### Chronic illnesses (Kuwait)

Shah et al. [28] provided community-based information on the prevalence of diabetes, hypertension and heart disease and their comorbidity. They analysed data from a cross-sectional household survey of 2,487 Kuwaiti nationals aged 50 and over in 2005/2006. Doctor-diagnosed prevalence of hypertension, diabetes and heart disease were reported to be 53.4, 50.6 and 17.5%, respectively. The prevalence of each of the three diseases increased linearly by age among both sexes and comorbidity of the three diseases increased from 3.6 to 9.4 and to 20.9% among those aged 50–59, 60–69 and ≥70 years, respectively. Using logistic regression, the authors showed that the prevalence of chronic illnesses was significantly higher among persons who were older, retired, non-Bedouin, less educated, had higher income, were less socially active, were obese and had poorer exercise behaviour. The prevalence of diabetes and heart disease was significantly lower among women than men [28].

### Diabetes and complications (Saudi Arabia)

Alwakeel et al. [29] conducted a study aiming to determine the prevalence of concomitant microvascular and macrovascular complications of diabetic nephropathy. They retrospectively reviewed the medical records of 1,952 type 2 diabetic patients followed-up at Security Forces Hospital, Riyadh, Saudi Arabia from January 1989 to December 2004. The authors found that concomitant diabetic complications included cataract (38.2%), acute coronary syndrome (36.1%), peripheral neuropathy (24.9%), myocardial infarction (24.1%), background retinopathy (22.4%), stroke (17.6%), proliferative retinopathy (11.7%), foot infection (7.3%), limb amputation (3.7%) and blindness (3%). Hypertension was documented in 577 (92.2%) patients, dyslipidemia in 266 (42.5%) and mortality from all causes in 86 (13.7%). There were 148 (23.6%) patients with one complication, 81 (12.9%) with two, 83 (13.3%) with three, and 61 (9.7%) with four or more. Complications were significantly more prevalent among males with greater number reaching end-stage renal disease level than females [29].

### Chronic complications and associated factors in type 2 diabetes (Pakistan)

Shera et al. [30] carried out a study to determine the prevalence of chronic complications and associated factors in type 2 diabetes in 500 diabetic patients aged 25 years or above, attending the clinic of Diabetic Association of Pakistan (DAP), Karachi. Every 5th registered diabetic patient was examined for the presence/absence of micro and macro vascular complications and associated factors. Age and sex were the socio-demographic characteristics considered. Of the 500 diabetic patients examined (160 males, 340 females), retinopathy was seen in 43%, neuropathy in 39.6% and foot ulcers in 4%. Nephropathy was found in 20.2%, and was significantly associated with hypertension. The prevalence of microvascular complications was significantly related to duration of diabetes, hypertension and obesity. Hypertension was manifest in 64.6% patients. Macrovascular complications were encountered in 102 diabetic patients, with angina in 85, heart attack in 25 and stroke in 13. The authors concluded that the prevalence of diabetic microvascular complications was higher in people with poor glycaemic control, longer duration of diabetes and associated hypertension and obesity. However, no analysis was given according the socio-demographic characteristics [30].

### Cardiovascular health among adults in Syria

In 2004, Maziak et al. [31] carried out a cross-sectional survey of adults 18–65 years old residing in Aleppo-Syria, involving 2038 household representatives. The socio-demographic variables considered were: age, sex, education, occupation, income and household density. The findings indicated an annual crude death rate due to CVD of 314 per 100,000, of these 179 were due to heart disease, and 135 due to stroke. More men died from heart disease than women, while the opposite was true for stroke. The authors stressed comorbidity with hypertension detected in 40.6% (47.7% men, 34.9% women), obesity in 38.2% (28.8% men, 46.4% women). Of those surveyed, 39.3% had one CVD risk factor, 27.4% had two risk factors, and 8.3% had three risk factors. Main predictors of clustering of risk factors were older age, male gender, and low education [31].

### Heart diseases, diabetes (Pakistan)

A hospital based cross-sectional study was conducted at the National Institute of Cardiovascular Disease in Karachi Pakistan in order to estimate the level of knowledge of modifiable risk factors of heart disease and determine the factors associated with good level of knowledge among 720 patients with acute myocardial infarction. The authors considered demographic and socio-economic characteristics (age, sex, ethnicity, marital status, literacy status, income) and comorbidity with diabetes and hypertension. Knowledge of four modifiable risk factors of heart disease: fatty food consumption, smoking, obesity and exercise were assessed. A mere 42% of the study population had a good level of knowledge. Independent predictors of "good" level of knowledge were: more than ten years of schooling verses no schooling at all (OR=2.5) and nuclear family system verses extended family system (OR= 2.54). In addition, Sindhi ethnicity (OR=3.03), higher level of exercise (OR=2.76) and non user of tobacco (OR=2.53) were also predictors of good level of knowledge [32].

### Metabolic syndrome, obesity, hypertension, diabetes (Jordan)

Zindah et al. [33] used a multistage random sampling to compare self-reported health information to actual medical measurements concerning comorbidity of obesity with diabetes, heart disease, asthma, hypertension and high cholesterol in Jordanian population. They used age, sex and education as socio-demographic characteristics. The study found that obesity was significantly associated with diabetes, high blood pressure, high cholesterol, and asthma. Compared with adults of normal weight, obese adults had a fully adjusted odds ratio (OR) of 3.27 for diabetes; 3.69 for high blood pressure; 3.45 for high cholesterol; and 5.12 for asthma. The logistic regression analysis showed that sex, age and education were not significantly associated with comorbid obesity [33].

### Diabetes, obesity, hypertension, cholesterol (Jordan)

Khader et al. [34] considered data of 1121 participants aged 25 years and above, randomly selected with the aim to determine the prevalence of obesity in northern Jordan, identify its associated factors, assess its association with selected comorbidities, and determine how the prevalence of obesity has changed in Jordan over 10 years. The socio-demographic characteristics (gender, age, marital status, education) as well as information on selected metabolic disorders and their potential risk factors were obtained. The age-standardized prevalence of obesity in northern Jordan was 28.1% for men and 53.1% for women. Irrespective of age or measure used, women always had a considerably higher prevalence of obesity than men. The prevalence of obesity varied greatly with age, generally increasing, irrespective of the measurement used. There has been a significant increase in the prevalence of obesity over a period of ten years for both men and women aged 60 years and above only. When important variables were taken into account in logistic regression analyses, obesity was significantly associated with increased odds of having all studied metabolic abnormalities. Female gender, increase in age, being married, former smoker or non smoker, and fewer than 12 years of education were significantly associated with increased odds of obesity. The authors concluded that the study demonstrated alarming rates of obesity and of its associated comorbidities among Jordanians, especially among women [34].

### Obesity and diabetes (Palestine)

The authors of a population-based survey of 485 (285 females, 190 males) Muslims living in the urban Palestinian community of Ramallah found that obesity prevalence was higher in women than in men (49% vs. 30%), while central obesity prevalence was higher in men (59% vs. 25%) than in women. Central obesity was significantly associated with hypertension (OR: 2.26) and diabetes (OR: 2.11) and the prevalence of comorbid obesity increased steadily with age in both sexes. No indication was given on the relationship of education with comorbidity. The authors of this study concluded that obesity and central obesity were prevalent in the urban Palestinian population and their association with diabetes, hypertension, and dyslipidemia point to a potential rise in CVDs [35].

### Obesity, metabolic syndrome (Iran)

A study of 984 middle-aged women in Babol, Iran, found a 31% prevalence of Metabolism Syndrome. Abdominal obesity was observed in 76.6% of participants. The prevalence of hypertension, high fasting blood glucose, high triglycerides, and low HDL-C were 12.1, 12.1, 41.5 and 48.6%, respectively. Increased risk for Metabolic Syndrome was associated with low education (OR: 2.78), older age (OR: 2.07) and an occupation of housekeeping (OR: 3.92) or farming (OR: 20.54) [36].

### Medical comorbidities after renal transplantation (Pakistan)

In order to assess the prevalence of medical comorbidities after kidney transplantation, Hollisaaz et al. [37] conducted a cross-sectional study on 119 kidney transplant recipients during 2006. Using Ifudu comorbidity index, they identified 14 chronic illnesses among patients undergoing maintenance hemodialysis. Eighty-three (90.4%) subjects had at least one medical comorbidity and the most frequent comorbidities were non ischemic heart diseases including hypertension (63%), visual disturbances (35.2%), low back pain and spine and joint disorders (25.21%), and musculoskeletal disorders (23.5%). The authors found that a higher score of comorbidity was significantly correlated with lower economic status, but not with age, gender, marital status, educational level, cause, or duration of end-stage renal disease [37].

### Pattern of acute renal failure in a tertiary hospital in the United Arab Emirates (UAE)

To evaluate the pattern, management and outcome of acute renal failure (ARF) in a tertiary hospital, Bernieh et al. [38] analysed the data of 81 patients admitted with or developing ARF in hospital between January 2002 and June 2003. The 45 men and 36 women were managed either on the ward (*n* = 48; 59%) and or in the ICU (*n* = 33; 41%). Comorbid conditions were hypertension (35%); diabetes (33%); chronic renal failure, (23%); ischemic heart disease (23%); and liver disease (15%). The most common predisposing factor was hypotension (52%), dehydration (40%), and drug nephrotoxicity (25%). This study showed that majority of ARF developed in-hospital with oncology patients constituting the greatest proportion. Sepsis was the leading cause of ARF and hypotension, the main predisposing factor. Patients treated in the ICU showed a worse prognosis for both patient and renal survival. Age, sex and ethnicity were the demographic data considered but no analysis was done [38].

Most of the WHO Eastern Mediterranean countries are engaged in a multidimensional transition (economic, demographic, geographic and epidemiological). People are living longer and consequently are more exposed to multimorbidity, especially with the increasing trend of non communicable diseases in the region [3]. In 2008, non communicable diseases caused 2.3 million deaths, representing 53% of the annual mortality in the WHO Eastern Mediterranean Region. Cardiovascular diseases, cancer, respiratory diseases and diabetes caused 55%, 14%, 9% and 5% of deaths respectively (Table 1). In 2009, Bokhari and Khan published a paper reviewing the knowledge on the burden and association of oral and systemic diseases [39]. The authors stressed that the association of oral diseases with NCDs such as diabetes, CVDs, CRD, osteoporosis and chronic renal failure was widely reported in the literature from developed countries but there was a crucial paucity of information and research in the WHO Eastern Mediterranean Region. In fact, of the 76 papers cited in their review, there was not a single citation from WHO Eastern Mediterranean Region dealing with association of oral diseases and NCDS. Another review published by Benamer et al. in 2010 on the epidemiology of headache in Arab countries included only four paper from Saudi Arabia(2), Oman(1) and Qatar(1) [40]. Unfortunately, no contribution on this topic fulfilled the criteria to be included in the present review.

Multimorbidity is an issue of growing importance for oncologists in developed countries [41]. However, despite the alarming increase of cancer incidence in Arab countries and the high cost of its treatment, generally unaffordable by disadvantaged populations, our review could hardly include one contribution from Pakistan [13]. Similarly, only two papers on respiratory diseases fulfilled the inclusive criteria [14,15].

The overlapping of NCDS makes it difficult to give a classification of the papers by diseases or group of diseases. Roughly, of the 26 papers included, nearly half (12) treated comorbidity of depression and mental diseases with NCDS [16-27]. Another set of 11 papers dealt with multimorbidity involving diabetes, CVDs, renal diseases, obesity and metabolic syndrome [28-38]. As indicated earlier, mutimorbidity is naturally associated with older age [13,17,19,20,23,25,27,28],[31,34,36]. One study in Qatar found that middle aged people were among the high-risk groups for depression and anxiety disorders [24]. Association of gender with multimorbidity indicated that women were more affected [17-19,21-25,35,36] than men [26,28,29].

**Figure 1:**
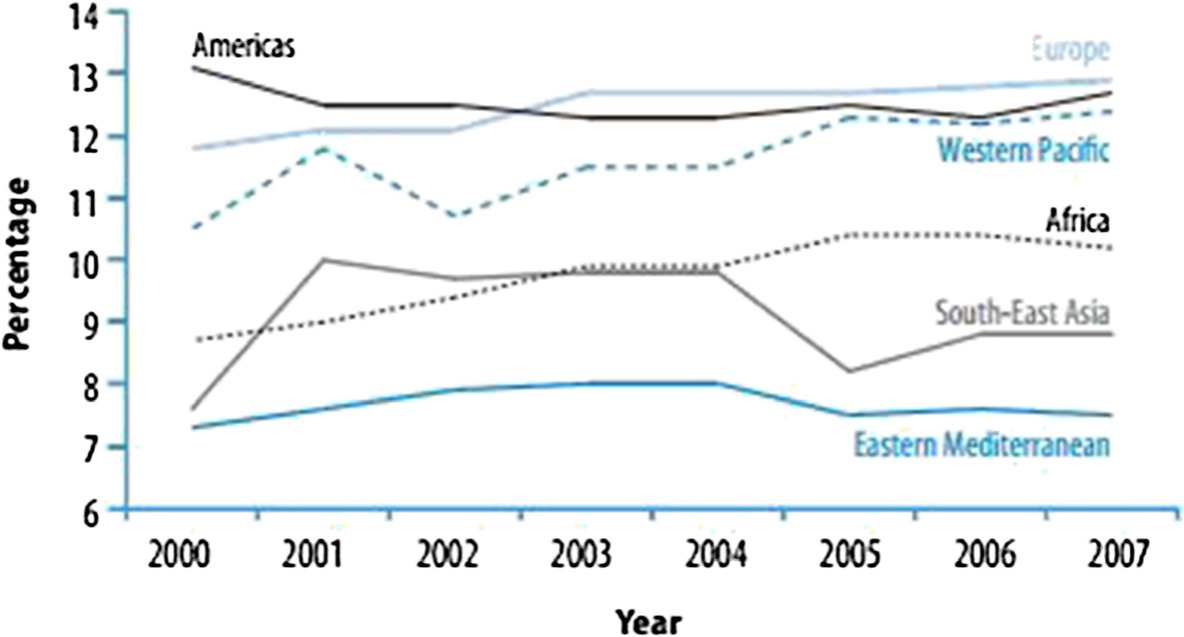
**Percentage of government expenditure on health as a percentage of total government expenditures by WHO region**^**2**^**, 2000–2007 **[43]** (Reproduced by kind permission of the World Health Organisation).**

Low economic status, generally measured by low income, was highly associated with mutimorbidity in many studies [13-18,26,37]. Of the 15 studies which considered association of education with multimorbidity, the majority found that low educated men and women were more likely to suffer from multimorbidity than people with higher level of education [13-15,17,18,21,26,28,31,34],[36]. Association with higher education was indicated in one study [24]. The association of occupation with multimorbidity showed that unemployed, house-keeping and retired people were more exposed [17,25,28,36]. Being employed was found to be associated with multimorbidity in one study [27]. Among studies which considered marital status few of them found association with mutimorbidity. Being married was seen to be significantly associated with comorobidities in Qatar [24] and Jordan [34] while association with being alone, divorced or widowed was indicated in three studies [17,23,25]. Ethnicity is an important social determinant of health in the gulf where nationals and non nationals (also said expatriates) have not the same accessibility/affordability of health services. However, although ethnicity was indicated in few studies, only a study in Pakistan found that Sindhi ethnicity was a predictor of good level of knowledge of modifiable risk factors of heart disease [32]. Finally, association of multimorbidity with milieu of residence was indicated in South Lebanon and Bekaa [14], Residence in formal in Syria [16], Kuwaiti non Bedouin [28] and urban Palestinian [35]. Cancer, CVDs and complications of diabetes (eye disease, kidney disease, amputations and foot disease) are the major causes of morbidity and mortality, resulting in high direct cost of care, high indirect cost in loss of productivity, and much societal stress [42]. These chronic diseases can quickly drive families into impoverishment by sucking up household resources. More generally, as indicated by the WHR2010 payment for health services pushes 100 million people into poverty each year worldwide and, out-of-pocket payments may represent more than 50% of the total health expenditures in low-and middle income countries [22]. As indicated by the Figure 1, among all WHO regions, governments of the WHO Eastern Mediterranean Region spend the least on health. Indeed, government expenditure on health as a percentage of total government expenditure is 3.8% in Egypt, 6.1 in Jordan, 6.3% in Kuwait, 3.6% in Lebanon, 5.4% in Morocco, 2% in Sudan, 4.5% in Syria, 7.7% in Tunisia, 7.7 in UAE: 7.7 and 4% in Yemen [43].

Consequently, individuals and household are obliged to highly contribute to medicines and treatments. Indeed, in 2006, out-of-pocket expenditure as % of total health expenditure accounted for 53.5% in Egypt, 38.1% in Jordan, 20.3% in Kuwait, 59.7% in Lebanon, 49.9% in Morocco, 63.4% in Sudan, 52.6% in Syria, 39.7% in Tunisia, 17.8% in UAE and 59.% in Yemen [43].

## Conclusion

Non communicable diseases are one of the biggest challenges facing health decision makers in the world and particularly in WHO Eastern Mediterranean countries where the multidimensional transition (economic, demographic, epidemiological and geographical) is boosting increases in multimorbidity of depression and mental diseases, cardiovascular diseases, diabetes, cancer and respiratory diseases among the whole population but with the highest burden among the least disadvantaged individuals or subpopulations. This review shows a noticeable lack and/or paucity of publications on multimorbidity and equity either geographically (no publication from North African countries) or thematically (no publication on headache and comorbidities). In the quasi totality of papers cited, socio demographic and socioeconomic variables were found to be associated with multimorbidity. Generally, older age, female gender, low education and low income people were seen to be likelier to suffer from multimorbidity. Health decision makers in WHO Eastern Mediterranean countries should pay a particular attention to the association between equity and multimorbidity and opt for cost effective strategies based on early diagnosis and sensitisation for healthy diet, physical activity, no smoking and no alcohol.

## Competing interests

The authors declare that they have no competing interest.
